# COVID-19 precautionary behavior: the Israeli case in the initial stage of the outbreak

**DOI:** 10.1186/s12889-020-09818-8

**Published:** 2020-11-16

**Authors:** Shiri Shinan-Altman, Inbar Levkovich

**Affiliations:** 1 The Louis and Gabi Weisfeld School of Social Work, Bar Ilan University, Ramat-Gan, 52900 Israel; 2 Faculty of Graduate Studies, Oranim Academic College of Education, Kiryat Tiv’on, Israel

**Keywords:** COVID-19, Emotional reactions, Knowledge about COVID-19, Precautionary behavior, Perceived susceptibility

## Abstract

**Background:**

The lay public’s behavioral responses during a virus spread, such as the COVID-19, play an important role in bringing the outbreak under control, and provide insights into development of risk communication messages to the public. Therefore, this study aimed to explore the association between knowledge about COVID-19, perceived susceptibility, emotional reactions and precautionary behavior among the Israeli lay public at the beginning of the COVID-19 outbreak.

**Method:**

A cross-sectional study was conducted among 1407 participants, aged 18 + .

Participants completed measures of knowledge about COVID-19, perceived susceptibility, emotional reactions, precautionary behavior, and socio-demographic questionnaires. A hierarchical regression model was calculated with precautionary behavior as the dependent variable.

**Results:**

Findings indicated that precautionary behavior was higher for females, older participants, participants with higher levels of knowledge about COVID-19, and participants with greater negative emotional reactions. A negative curvilinear relationship was found between perceived susceptibility and precautionary behavior, so that the latter was highest for participants with moderate perceived susceptibility. All interaction terms were non-significant.

**Conclusions:**

Findings suggest that in order to enhance precautionary behavior in the initial stage of a virus outbreak, it is recommended to pay attention to the public’s knowledge about the virus, perceived susceptibility and emotional reactions. Although negative feelings about the virus may motivate preventive behavior, it is important to address these feelings.

**Supplementary Information:**

The online version contains supplementary material available at 10.1186/s12889-020-09818-8.

## Background

*Coronavirus disease 2019* (COVID-19) is an infectious disease caused by SARS-CoV-2 [1]. The average incubation period for COVID-19 was estimated to be about 5.1 days [2]. The most common clinical symptoms are cough and fever, in addition to other non-specific symptomatology, such as dyspnea, fatigue, muscle soreness and headache [3]. Most people contracting the virus will experience mild to moderate respiratory illness and recover without particular treatment [4, 5]. However, older adults, and those with underlying medical problems - such as diabetes, cardiovascular disease, cancer and chronic respiratory disease - are more likely to develop a serious illness [4, 6].

First appearing in December 2019 in China, COVID-19 has rapidly spread around the globe [7], including Israel. The first Israeli person with COVID-19 was diagnosed on February 21, 2020. Since then, in Israel, thousands have been isolated in their homes, hundreds have been diagnosed and, as of May 11, 2020, 254 individuals have died from this virus. The Israeli Ministry of Health [8] continues to release updated guidelines, instructions and restrictions for the lay public regarding how to behave in the new daily routine. These guidelines include precautionary behavior such as washing hands often with soap and water or an alcohol-based hand sanitizer; avoiding close contact with people with symptoms such as coughing or sneezing; avoiding shaking hands; and covering one’s mouth and nose when coughing or sneezing [8]. However, as Israeli citizens receive information about COVID-19 from various sources, they may have incorrect knowledge regarding the disease (e.g., regarding which populations are at a high risk of contracting the virus). In addition, the numbers of suspicious and diagnosed cases continue to increase, which may affect public’s perceptions about the severity and controllability of the virus [1, 9].

Among the factors that may influence willingness and motivation to adopt precautionary behaviors are perceived susceptibility [10] and emotional reactions such as worry, fear and stress [11]. *Perceived susceptibility* has been used to measure one’s perception of the likelihood of contracting a disease or a virus [12]. *Worry* is assumed to involve an emotional process and is closely related to anxiety [13]. *Fear* is defined as an unpleasant emotional state that occurs in response to a real threat or danger [14]. Regarding *stress,* studies on severe acute respiratory syndrome (SARS) indicated that the outbreak of an unprecedented virus can cause immense stress to the lay public and influence the public’s precautionary behaviors [11, 15].

Knowledge about COVID-19, perceived susceptibility, emotional reactions and precautionary behaviors among the lay public are significant in the control of epidemics, as was learned after SARS [16]. Both cognitive (e.g., knowledge about COVID-19, perceived susceptibility) and emotional matters (e.g., worry, fear and stress) have an important bearing on coping with health threats [17]. However, research literature regarding these concepts in the area of COVID-19 is only now beginning to emerge [9]. Therefore, the aim of the current study was to examine the association linking between knowledge about COVID-19, perceived susceptibility, emotional reactions and precautionary behavior. This aim is especially important, given that the behavior of the general population can play an important role in both the spread and control of infectious diseases [18].

## Methods

### Procedure and participants

Prior to commencing the study, authorization was obtained from Bar-Ilan University’s Ethics Committee (approval No. 032003). To minimize personal contact during the outbreak, the questionnaires were administered through the Qualtrics online platform (www.qualtrics.com) on different social media outlets. The survey was distributed to the public using two main social media platforms: Facebook and WhatsApp. In the first stage, intensive sampling was accomplished through social media and social networks platforms. In the second stage, snowball sampling was performed to reach more circles of participants.

The sample size was calculated with G*Power (ver.3.1 [19]). Considering high power of 0.95, an alpha level of 0.05, and a low effect size of f^2^ = 0.02, a regression with six predictors requires 1050 participants. Raising the effect size to a moderate level of f^2^ = 0.15, a regression with six predictors requires 146 participants. Overall, a total of 1435 Israelis visited the online survey between March 12, 2020 and March 21, 2020. Inclusion criteria for the study were: (1) individuals aged 18+; and (2) Hebrew speakers (about 84% of people in Israel speak Hebrew in a high level [20]. Exclusion criteria were: (1) minors (under the age of 18) (*n* = 14); and (2) responses to the items in a similar pattern (e.g., choosing the same answer across multiple consecutive items or within the whole questionnaire) or not completing the questionnaire in its entirety (*n* = 14). Inclusion and exclusion criteria were applied after people participated in the survey. Measures were taken to avoid having participants respond multiple times, such as using the “Prevent Ballot Box Stuffing” feature on Qualtrics.

### Measures

*Precautionary behavior* was measured using a 4-item scale created by the authors following the precautionary guidelines issued by the Israeli Ministry of Health [8]. The scale’s validity was reached by expert validity, a form of content validity. In this validity process, the scale is reviewed by a panel of four expert physicians in order to eliminate totally irrelevant items from the instrument [21] and to re-phrase or supply new wording for items related to the measured construct where necessary [22]. Participants were asked to indicate how often they perform various precautionary behaviors on a 5-point scale (from 1 = not at all to 5 = very often) (e.g., “How often do you wash your hands”?). A composite index of the average of all items was created, a higher score indicating that participants display more precautionary behavior. Sample items include washing hands with soap and water or alcohol-based hand sanitizer, and avoiding close contact with people with symptoms such as coughing or sneezing. The internal consistency of the index was moderate (Cronbach’s α = 0.75).

*Knowledge about COVID-19* was measured using a 6-item COVID-19 knowledge test assessing the symptoms, diagnosis, risk factors, ways of transmitting the virus, ways to protect oneself from contracting COVID-19 and knowledge regarding where to refer a person who is suspected of having contracted COVID-19 (e.g., “To what extent can you identify the symptoms of COVID-19?”). The scale’s validity was reached using expert validity (as detailed in the *Precautionary behavior* measure). Answers were rated on a 5-point Likert-type scale, ranging from 1 = don’t know at all to 5 = know very much. A composite index of the average of all items was created, with a higher score indicating higher levels of knowledge about COVID-19. The internal consistency of the index was very good (Cronbach’s α = 0.82).

*Perceived susceptibility* was assessed based on previous studies conducted among the general public [13], with a single- item measure examining the extent to which the participant thinks he/she will contract the virus. “How likely do you think it is that you will contract COVID-19?” Answers were rated on a 5-point Likert-type scale, ranging from 1 = not at all likely to 5 = very likely.

*Emotional reactions towards COVID-19* were assessed based on previous studies conducted among the general public [13], with 3 items concerning worry, fear and stress as a result of COVID-19 (e.g., “How much do you worry about COVID-19?”; “How much are you afraid of COVID-19?”). Answers were rated on a 5-point Likert-type scale, ranging from 1 = not at all to 5 = very much. A composite index of the average of all items was created, with a higher score indicating higher levels of negative emotional reactions towards COVID-19. The internal consistency of the index was excellent (Cronbach’s α = 0.94).

*Socio-demographic variables* included gender, age, years of education, marital status (married/divorced/widow/single/other), number of children, medical problems (yes/no), health status (bad/reasonable/good), home isolation since the outbreak of COVID-19 (yes/no), diagnosed with COVID-19 (yes/no), resources that can make it easier to cope with COVID-19 (more information regarding COVID-19/professional support/non-professional support/working from home/other).

All measures are presented in Additional file 1.

### Statistical analyses

Data were analyzed using SPSS ver. 25. Descriptive statistics were used to describe the participants’ demographic characteristics and the research variables. Pearson correlations were calculated to assess the associations linking the research variables. Strength of correlation was as follows: 0**–**0.20, weak; 0.21**–**0.50, moderate; 0.51**–**0.80, good; and 0.81**–**1.00, excellent. A hierarchical regression model was calculated with precautionary behavior as the dependent variable. Gender (1-male, 0-female) and age were entered in the first step, knowledge about COVID-19 in the second, perceived susceptibility in the third, and negative emotions in the fourth. As perceived susceptibility had a curvilinear relationship with precautionary behavior, all variables were standardized.

## Results

As can be seen in Table 1, the study included 1407 participants. The majority of the respondents were female (80%), with a mean age of about 41 years (range 18–97) and an average education of approximately 16.5 years (range 9–30). Most were married (63%) with a mean of two children. About 85% did not have health problems and about 80% reported good health status. Only 5% reported being at home in isolation since the outbreak of COVID-19 and the most favorable resource that participants reported which could ease their coping with COVID-19 was working from home.

**Table 1:** Participants’ characteristics (*N* = 1407**)**

Socio-demographic characteristics	
Gender (%)	
Male	282 (20.1)
Female	1119 (79.9)
Mean age (SD), range	40.97 (14.66), 18–97
Mean number of years of education (SD), range	16.44 (3.66), 9–30
Marital status (%)	
Married	879 (62.7)
Divorced	81 (5.8)
Widow	24 (1.7)
Single	380 (27.1)
Other	37 (2.6)
Mean number of children (SD), range	2.18 (1.39), 0–9
Health problems (%)	
Yes	214 (15.3)
No	1186 (84.7)
Health status (%)	
Bad	19 (1.4)
Moderate	276 (19.6)
Good	1108 (79.0)
Home isolation since the outbreak of COVID-19 (%)	
Yes	70 (5.0)
No	1333 (95.0)
Diagnosed with COVID-19	
Yes	0
No	100
Resources that can make it easier to cope with COVID-19 (%)	
More information regarding COVID-19	260 (19.4)
Professional support	172 (12.8)
Non-professional support	143 (10.7)
Working from home	529 (39.4)
Other	237 (17.7)

**Fig. 1:**
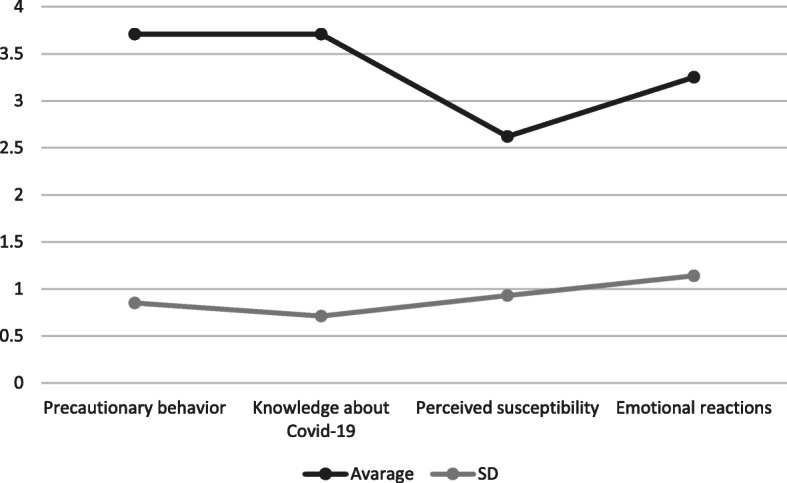
Means and SDs of study’s variables

Table 2 summarizes the means, SDs, ranges and correlates of the study variables. As can be seen, the mean score for precautionary behavior was 3.71 (SD = 0.85), in a range of 1**–**5, meaning the precautionary behavior score was relatively high. The mean score for knowledge about COVID-19 was also relatively high, while the means scores for emotional reactions and perceived susceptibility were moderate. In other words, participants in the current study had relatively high knowledge about COVID-19, believed there was a moderate probability they would contract COVID-19, and had moderate negative feelings as a result of the virus. Figure 1 summarizes the means and SDs of the study’s variables.

**Table 2:** Correlates, Means, SDs, and ranges of study variables (*n =* 1407)

Variables	Precautionary behavior	Knowledge about COVID-19	Perceived susceptibility	Emotional reactions
Precautionary behavior	–			
Knowledge about COVID-19	*r =* 0.41 *p =* 0.000	**–**		
Perceived susceptibility	*r =* 0.07 *p =* 0.01	*r =* 0.04 *p =* 0.19	**–**	
Emotional reactions	*r =* 0.24 *p =* 0.000	*r =* −0.03 *p* = 0.28	*r =* 0.31 *p =* 0.000	**–**
Mean	3.71	3.71	2.62	3.25
SD	0.85	0.71	0.93	1.14
Possible range	1–5	1–5	1–5	1–5
Actual range	1–5	1–5	1–5	1–5

According to Table 2, positive associations were found linking knowledge about COVID-19 and emotional reactions with precautionary behavior. In other words, the more knowledge participants had about COVID-19, and the more negative emotions they had towards COVID-19, the more precautionary behavior they exhibited. In addition, higher perceived susceptibility was associated with higher levels of negative emotions towards COVID-19.

Precautionary behavior was higher among females (Mean = 3.74, SD = 0.84) than males (Mean = 3.57, SD = 0.88) (t(1399) = − 3.02, *p* = 0.003), and higher among married participants (Mean = 3.78, SD = 0.79) than non-married participants (Mean = 3.58, SD = 0.92) (t(964.38) = 4.15, *p* < 0.001). The correlation between age and precautionary behavior was significant (*r =* 0.24, *p <* 0.001), as was the correlation between number of children and precautionary behavior (*r =* 0.12, *p* < 0.001). In addition, the correlation between years of education and precautionary behavior was also found to be significant (*r =* 0.19; *p* < 0.001). Finally, precautionary behavior was higher among participants with health problems (Mean = 3.91, SD = 0.83) than participants with no health problems (Mean = 3.67, SD 0.85) (t(1398) = 3.82, *p* < 0.001). Thus, analyses were calculated while controlling for gender and age (age was highly interrelated with marital status *r =* 0.35, *p <* 0.001, number of children *r =* 0.52, *p <* 0.001, and years of education *r =* 0.42, *p <* 0.001).

### Regression analysis for identifying precautionary behavior correlates

A hierarchical regression model was calculated for precautionary behavior, with gender, age, knowledge about COVID-19, perceived susceptibility, and emotional reactions as predictors. A preliminary analysis of precautionary behavior by perceived susceptibility revealed that, for very low and low susceptibility, the means for precautionary behavior increased from 3.49 (SD = 0.98) to 3.65 (SD = 0.83); for moderate susceptibility, the mean for precautionary behavior was even higher 3.81 (SD = 0.79); and for high and very high susceptibility the means decreased to 3.69 (SD = 0.82) and 3.62 (SD = 1.15), respectively. Indeed, the relationship was found to be curvilinear-quadratic (contrast estimate = − 0.20, SE = 0.09, *p* = 0.033, 95%CI -0.38, − 0.02). Thus, the regression model was defined to test the curvilinear-quadratic effect of perceived susceptibility on precautionary behavior. For this purpose, both perceived susceptibility and its squared value were entered into the regression model, since a significant contribution of the squared variable, beyond the original variable, establishes the curvilinear effect.

The results presented in Table 3 reveal significant models, with 27% of the variance in precautionary behavior being explained in the final model. Precautionary behavior was higher for females, older participants, participants with higher levels of knowledge about COVID-19, and participants with greater negative emotional reactions. A negative curvilinear relationship was found between perceived susceptibility and precautionary behavior; the latter was highest for participants with moderate perceived susceptibility, and lower for participants with low and high perceived susceptibility. All interaction terms were non-significant.

**Table 3:** Regression analysis for precautionary behavior (*N* = 1181)

	Model 1	Model 2	Model 3	Model 4
Gender - male	β = −0.15*** *p =* 0.01	β = −0.12*** *p =* 0.01	β = − 0.11*** *p* = 0.02	β = − 0.06* *p =* 0.03
Age	β = 0.26*** *p =* 0.01	β = 0.19*** *p =* 0.000	β = 0.19*** *p* = 0.000	β = 0.23*** *p =* 0.000
Knowledge		β = 0.36*** *p =* 0.01	β = 0.35*** *p =* 0.000	β = 0.35*** *p =* 0.000
Perceived susceptibility			β = 0.06* *p =* 0.000	β = −0.02 *p =* 0.01
Perceived susceptibility- squared			β = −0.07* *p =* 0.04	β = −0.07* *p =* 0.02
Negative emotions				0.29*** *p =* 0.01
**Adj.R**^**2**^	0.073 *p =* 0.01	0.193 *p =* 0.01	0.200 *p =* 0.000	0.272 *p =* 0.000

F(6, 1174) = 74.39, *p* = 0.001, *p < 0.05, ***p < 0.001

## Discussion

The lay public’s behavioral responses during a virus outbreak play an important role in bringing the outbreak under control, and provide insights into the development of risk communication messages to the public [23, 24]. Therefore, this study aimed to explore the associations linking knowledge about COVID-19, perceived susceptibility, and emotional reactions with precautionary behavior among the Israeli lay public at the beginning of the COVID-19 outbreak.

Overall, our findings indicate that the precautionary behavior score was relatively high among participants. This is an encouraging finding, given that the spread of this virus depends on everyday human behaviour, i.e., hygiene behaviours and social contact [25]. The current study was conducted in the fourth week of the epidemic in Israel, indicating that the Israeli public began to address the basic requirements for precautionary behavior within a relatively short time period (e.g., washing hands often with soap and water or an alcohol-based hand sanitizer). It should be noted that during the outbreak of COVID-19, all types of Israeli media were used to provide current and comprehensive information about world infection trends and Israel’s situation. These various channels of access to information are relatively easy to follow and may have served as significant sources of preventive actions. This strategy of spreading information regarding the virus and its risk factors may also explain the association we found between knowledge about COVID-19 and precautionary behaviour.

Our results indicate that negative curvilinear relationship was found between moderate perceived susceptibility and precautionary behavior. It seems that, at least during the initial phase of the outbreak, perceived susceptibility of contracting the virus was associated with the potential risk attributed to one’s own individual behaviors [26]. However, perhaps the reason we found only moderate levels of perceived susceptibility to be associated with precautionary behavior was because, in this initial stage of the virus spread, it was difficult for people to consider the possibility of contracting the virus. Furthermore, during this initial period, people might have engaged in “wishful thinking” beliefs (e.g., “it won’t happen to me”). The findings also indicate that lower precautionary behavior was associated with lower and higher perceived susceptibility. Namely, participants who believed they were less likely to become infected with COVID-19 performed less precautionary behavior. Interestingly, participants who believed they had a higher chance of contracting the virus also performed less precautionary behavior, perhaps because they thought that this behavior would not protect/help them.

In our sample, negative emotional reactions were associated with precautionary behavior. In other words, in the early stages of a crisis, the public may develop a protective response because of worry, fear and stress [27]. This finding indicates that in an acute threat situation, like COVID-19, the emotional aspects need to be taken into account; their significance must be recognized, and an adequate response should be provided [23]. In Israel, with the understanding that the virus was spreading, various organizations began to provide reactive and proactive emotional support to people in isolation, older people, and those seeking mental support on their own initiative.

This study has several limitations. First, as the survey was conducted during the fourth week of the virus outbreak, it presents an immediate and initial picture of the lay public’s reactions towards COVID-19. However, with the spread of the virus, the behavioral guidelines are constantly changing; therefore, it is advisable to continue to explore preventive behaviors among the lay public across time. Second, we used a correlational design, which limits our capacity to demonstrate causal relationships. Third, the sample was not randomly selected, and thus may have been subject to bias. Indeed, female participants with higher education were over-represented in the sample, which makes the findings not entirely generalizable to other populations. Finally, participants were not asked if there was an elderly member within their family and they were not asked if someone in their family was infected by COVID-19.

## Conclusions

In order to enhance precautionary behavior in the initial stage of a virus outbreak, it is recommended to pay close attention to the public’s knowledge about the virus, perceived susceptibility and emotional reactions. Although negative feelings about COVID-19 may well motivate individuals to engage in preventive behaviors, it is important to address and legitimize these feelings. Therefore, ongoing scrutiny and monitoring of psychological outcomes resulting from epidemic outbreaks, and providing prompt targeted mental health interventions should become standard procedures in preparedness efforts worldwide.

## Supplementary Information

**Additional file 1.** Study’s survey. Includes all measures used in the study.

## Data Availability

The data that support the findings of this study are available from the authors upon reasonable request.

## Abbreviations

COVID-19: Coronavirus disease 2019
SARS-CoV-2: Severe acute respiratory syndrome coronavirus 2
SD: Standard deviation
