# Supplementary material for: COVID-19 precautionary behavior: the Israeli case in the initial stage of the outbreak
Source: BMC Public Health. 2020 Nov 16;20:1718. doi: 10.1186/s12889-020-09818-8 (PMC7668024; doi:10.1186/s12889-020-09818-8)
Supplement: Supplementary file 1 — Additional file 1. Study’s survey. Includes all measures used in the study. [file 12889_2020_9818_MOESM1_ESM.docx]

**The survey questionnaire**

**Please indicate how often you perform the following precautionary behaviors:**

|  | **Not at all** |  |  |  | **Very often** |
| --- | --- | --- | --- | --- | --- |
| Wash your hands with soap and water or an alcohol-based disinfectant | 1 | 2 | 3 | 4 | 5 |
| Avoid close contact with people suffering from symptoms such as sneezing cough or runny nose | 1 | 2 | 3 | 4 | 5 |
| Avoid shaking hands | 1 | 2 | 3 | 4 | 5 |
| Make sure to cough and sneeze into the elbow or disposable handkerchief | 1 | 2 | 3 | 4 | 5 |

**Regarding the following sentences, please rate the answers from 1 – don’t not at all to 5 - to know very much.**

| **How much do you know...** | **don’t know at all** |  |  |  | **know very much** |
| --- | --- | --- | --- | --- | --- |
| What are the ways of infection with COVID-19? | 1 | 2 | 3 | 4 | 5 |
| What are the symptoms of COVID-19? | 1 | 2 | 3 | 4 | 5 |
| How COVID-19 is diagnosed? | 1 | 2 | 3 | 4 | 5 |
| Which people are at high risk of contracting COVID1-19? | 1 | 2 | 3 | 4 | 5 |
| What can be done to avoid contracting COVID-19? | 1 | 2 | 3 | 4 | 5 |
| Where to refer a suspected person who fell ill with COVID-19? | 1 | 2 | 3 | 4 | 5 |

**How likely do you think it is that you will contract COVID-19?**

| **not at all likely** |  |  |  | **very likely** |
| --- | --- | --- | --- | --- |
| 1 | 2 | 3 | 4 | 5 |

**Regarding the following sentences, please rate the answers from 1 - very low to 5 - very high.**

|  | **not at all** |  |  |  | **Very much** |
| --- | --- | --- | --- | --- | --- |
| How much do you worry about COVID-19? | 1 | 2 | 3 | 4 | 5 |
| How much are you afraid of COVID-19? | 1 | 2 | 3 | 4 | 5 |
| How much you are under pressure from COVID-19? | 1 | 2 | 3 | 4 | 5 |

**Finally, we would appreciate it if you could fill in the following details:**

Gender: ___________

Age: ___________

Years of education: __________

Marital status: 1. Married 2. Divorced 3. Widow 4. Single 5. other

Number of children: ________

Medical problems: 1. Yes 2. No

Health status: 1. Bad 2. Reasonable 3. Good

Home isolation since the outbreak of COVID-19: 1. Yes 2. No

Were you diagnosed with COVID-19? 1. Yes 2. No

Resources that can make it easier for you to cope with COVID-19:

1. More information regarding COVID-19 2. Professional support 3. Non-professional support 4. Working from home 5. Other.
